# The relationship between impostor phenomenon and emotional exhaustion among Chinese nurses: the mediating role of bi-directional work-family conflict

**DOI:** 10.3389/fpubh.2024.1410452

**Published:** 2025-01-15

**Authors:** Yuan Li, Fangxinrui Qiu, Biru Luo, Yanling Hu, Jie Li, Ying Xin Li, Ping He, Jinbo Fang

**Affiliations:** ^1^Department of Nursing, West China Second University Hospital, Sichuan University, Chengdu, China; ^2^Department of Pediatrics, West China Second University Hospital, Sichuan University, Chengdu, China; ^3^Key Laboratory of Birth Defects and Related Diseases of Women and Children (Sichuan University), Ministry of Education, Chengdu, China; ^4^The International Medical College of Chongqing Medical University, Chongqing, China; ^5^West China Hospital/West China School of Nursing, Sichuan University, Chengdu, China; ^6^Department of Nursing, The People’s Hospital of Jianyang City, Jianyang, China

**Keywords:** impostor phenomenon, emotional exhaustion, work–family conflict, Chinese nurses, mediation effect analysis

## Abstract

**Aims:**

The study aims to explore the relationship between impostor phenomenon and emotional exhaustion among nurses and to examine the potential mediating role of bi-directional work–family conflict.

**Methods:**

A cross-sectional survey using convenience sampling was conducted from January to April 2023, involving 4,088 Chinese nurses. Of those, 3,977 nurses across 43 public hospitals completed the web-based survey that included a sociodemographic information questionnaire, the short Clance Impostor Phenomenon Scale, the Bi-directional Scale of Work–Family Conflict, and the Emotional Exhaustion Scale. SPSS with Hayes’s PROCESS v4.2 Macro was employed to examine the mediation model using bootstrap techniques.

**Results:**

After controlling for confounding factors, impostor phenomenon was found to have a direct positive effect on emotional exhaustion (𝛽 = 0.134, 95% CI [0.122 to 0.145]); the two dimensions of work–family conflict, work interfering with family (𝛽 = 0.099, 95% CI [0.090 to 0.109]) and family interfering with work (𝛽 = 0.017, 95% CI [0.012 to 0.022]), served as parallel mediators in the relationship between impostor phenomenon and emotional exhaustion. Compared to family interfering with work, impostor phenomenon had a greater influence on emotional exhaustion through the mediation of work interfering with family, with a difference in the mediating effects of 0.082 (95% CI [0.073 to 0.096]). (The symbol *β* denotes the regression coefficient, estimated through mediation analysis using a bias-corrected bootstrapping procedure. CI represents the confidence interval for the specified parameter).

**Conclusion:**

This study reveals that impostor phenomenon not only directly affects emotional exhaustion but also exerts parallel mediation effects through bi-directional work–family conflict, with work interfering with family exerting a stronger mediating effect than family interfering with work. The findings elucidate the complex interplay between impostor phenomenon, an intrapersonal psychological factor, and work–family conflict, an interpersonal stressor, in contributing to emotional exhaustion among Chinese nurses, providing valuable insights to guide efforts aimed at safeguarding nurses’ mental health and well-being.

## 1 Introduction

Nurses play a pivotal role in delivering quality patient care, yet they are frequently plagued by high levels of occupational stress and burnout. In China, a nationwide survey revealed that over a third of nurses (33.8%) suffer from emotional exhaustion (1), a rate three times higher than the global average (11.23%) (2). Emotional exhaustion, a core component of burnout, has been associated with numerous negative consequences, such as reduced job satisfaction, impaired quality of care, and increased intention to leave and actual turnover (3–5). The prevalence of turnover intention among Chinese nurses has reached an alarming 42.8% (6). In response to this pressing concern, the Chinese government has implemented a series of policies and initiatives to promote nurses’ well-being and mitigate burnout. The “Healthy China 2030″ blueprint, launched in 2016, emphasized the imperative to enhance the working environment and mental health of healthcare professionals^1^. Subsequently, the “Nursing Development Plan (2021–2025)” further reinforced the importance of safeguarding nurses’ occupational health and preventing job burnout^2^. These policy measures underscore the critical need for effective strategies to support nurses’ well-being and alleviate emotional exhaustion.

While previous studies have investigated the impact of work-related stressors and individual characteristics on nurses’ emotional exhaustion (1, 7, 8), research into the psychological mechanisms underpinning this phenomenon, particularly those incorporating both intrapersonal and interpersonal factors, remains limited. As the largest nursing workforce in the world^3^, Chinese nurses’ well-being and retention have significant implications for global healthcare delivery. Therefore, unraveling the mechanisms that shape Chinese nurses’ emotional exhaustion is crucial for formulating evidence-based strategies to promote nurse well-being and mitigate burnout, which in turn has far-reaching implications for the quality and stability of healthcare systems worldwide.

## 2 Background

One intrapersonal factor that has gained emerging attention in the nursing literature is impostor phenomenon^4^. Impostor phenomenon refers to the persistent belief that one’s success is undeserved and the fear of being exposed as a fraud, despite objective evidence of competence (9). Individuals with impostorism often exhibit a range of interconnected traits including the impostor cycle, perfectionism, superheroism, atychiphobia (fear of failure), achievemephobia (fear of success), and denial of competence (9). For example, these individuals may find themselves trapped in the impostor cycle, a self-perpetuating pattern of anxiety, overwork, and self-doubt (10). Perfectionism prompts them to set unattainably high goals, leading to self-criticism over any perceived shortfall (11). This maladaptive perfectionism can engender a vicious cycle of overexertion and potential burnout, as individuals strive to prove their worth with “superheroic efforts” (11). Moreover, the constant fear of making mistakes, being exposed as incompetent, or facing higher expectations upon success can trigger chronic anxiety and stress, further depleting emotional reserves (9, 12). Impostorism also leads to a denial of competence, where individuals attribute their successes to external factors, thereby fuelling feelings of recurrent self-doubt and intellectual phoniness (9). These behavioral patterns render individuals with impostorism particularly prone to mental health disorders, such as burnout, depression, and anxiety, and can exacerbate other psychological conditions (10, 13).

Within nursing populations, the prevalence of impostor phenomenon is notably high, with estimates ranging between 36 and 75% (14). This heightened prevalence among nurses may be attributed to their unique professional context: the requirement for continuous competency development, high-stakes decision-making responsibilities, and the constant pressure to maintain impeccable performance in life-or-death situations (14, 15). Impostor phenomenon manifests uniquely in nursing practice, where the need for precise clinical judgment intersects with the emotional demands of patient care. Nurses experiencing impostorism often doubt their clinical competence despite demonstrated expertise, leading to excessive double-checking of decisions and emotional detachment as coping mechanisms (14, 15). According to the Conservation of Resources (COR) theory (16), this persistent self-doubt and hypervigilance depletes nurses’ psychological reserves, directly contributing to emotional exhaustion. This proposition aligns with the Job Demands-Resources (JD-R) model (17), which posits that an imbalance between job demands and personal resources can precipitate burnout. As impostorism consumes nurses’ cognitive and emotional resources, it may intensify the perceived demands of their work environment, thereby increasing the risk of exhaustion (14). Empirical studies have corroborated these theoretical assertions, demonstrating significant associations between impostor phenomenon and emotional exhaustion among nursing populations (14, 15, 18, 19) and other healthcare professionals (20, 21). Consequently, impostor phenomenon could be identified as a potent intrapersonal factor that contribute to emotional exhaustion and other adverse psychobehavioral outcomes among nurses, yet the pathways through which this relationship manifests remain poorly understood.

In addition to intrapersonal factors like impostor phenomenon, interpersonal factors such as work–family conflict have also been pinpointed as significant contributors to emotional exhaustion, with both work interfering with family (WIF) and family interfering with work (FIW) showing significant associations (22–25). Work–family conflict occurs when the demands of work and family roles are incompatible (26), a scenario frequently encountered by nurses due to the profession’s high demands and irregular schedules. In fact, over 40% of nurses have reported experiencing high levels of work–family conflict (25, 27). Nurses grappling with impostorism may find their WIF exacerbated, as they tend to overcommit to work in an attempt to validate their competence (28). On the other hand, the deep-seated self-doubt and psychological strain associated with impostor phenomenon can also undermine their family functionality, thereby elevating FIW (29). Accordingly, both WIF and FIW could serve as critical pathways through which impostor phenomenon contributes to emotional exhaustion in nurses. However, existing studies have not explicitly explored how impostor phenomenon interacts with work–family conflict to influence emotional exhaustion.

Moreover, the primary behavioral patterns associated with impostorism, such as excessive dedication to work and prioritizing occupational obligations, may lead to a greater spillover of work-related stress into the family domain than vice versa (30, 31). Reichl et al.’s meta-analysis (32) further revealed that the relationship between work–nonwork conflict and emotional exhaustion was more potent than that between nonwork–work conflict and emotional exhaustion, a trend observed among working adults from different cultural backgrounds. These findings resonate with the JD-R model (17), which highlights job demands as the foremost catalysts for exhaustion, with emotional resources being predominantly depleted by occupational rather than family demands. Based on the empirical and theoretical underpinnings, WIF may serve as a more salient mediator than FIW in the relationship between impostor phenomenon and emotional exhaustion.

Taken together, we proposed the following three research hypotheses:

*H1*: Impostor phenomenon has a direct positive effect on emotional exhaustion.

*H2*: Bi-directional work–family conflict, specifically WIF and FIW, parallelly mediates the relationship between impostor phenomenon and emotional exhaustion.

*H3*: The mediating effect of WIF is stronger than that of FIW in the relationship between impostor phenomenon and emotional exhaustion.

## 3 Methods

### 3.1 Aims

The current study aims to explore the relationship between impostor phenomenon and emotional exhaustion among a large multicenter sample of Chinese nurses and examine the potential parallel mediating roles of the two dimensions of work–family conflict.

### 3.2 Design

A multicenter cross-sectional design was employed in this study, adhering to the Strengthening the Reporting of Observational Studies in Epidemiology (STROBE) guidelines (33).

### 3.3 Settings and participants

Participants were recruited from 43 public hospitals, primarily located in the western and middle regions of China. Public hospitals serve as the primary healthcare service providers in China. The western and middle regions are comparatively less developed than the eastern and southern regions. Participants were eligible to participate if they met the following inclusion criteria: (i) being an active registered nurse; (ii) having over 1 year of experience in clinical nursing, and (iii) voluntarily agreeing to participate. Nurses on rotation, intern nurses, and nurses who had taken continuous leave for more than 6 months within the last year were excluded.

### 3.4 Measurements

#### 3.4.1 Demographic questionnaire

The study employed a succinct questionnaire to collect demographic and occupational details from the participating nurses. This questionnaire solicited information regarding their gender, age, marital status, educational level, working experience, and professional title. Additionally, to evaluate the socioeconomic status of the participants, we posed a single question: ‘Considering your income, education, and occupation, where would you rank your family within the broader social hierarchy?’ Responses were gaged on a 10-point scale, with 1 being the lowest and 10 being the highest position.

#### 3.4.2 Impostor phenomenon

Impostor phenomenon was assessed using the short Clance Impostor Phenomenon Scale (CIPS-10) (34). This version is a refined adaptation of the full-length CIPS (35), by selecting 10 essential, nonredundant items and incorporating important enhancements (34). An illustrative item is, “I’m afraid people important to me may find out that I’m not as capable as they think I am.” The CIPS-10 consists of 10 items rated on a 7-point frequency scale from ‘1 = never’ to ‘7 = always’ (34). The total score on the scale ranges between 10 and 70, where higher scores correspond to more severe instances of the impostor phenomenon. The CIPS-10 demonstrated excellent reliability in our study with a Cronbach’s alpha coefficient of 0.974.

#### 3.4.3 Work–family conflict

Work–family conflict was appraised using the Bi-directional Scale of Work–Family Conflict (36, 37). The scale consists of 6 items measuring work interfering with family (WIF; e.g., my job keeps me from spending time with my family members), and another 5 items measuring family interfering with work (FIW; e.g., my family demands make it hard for me to do my job well) (37). Participants’ responses were collected on a 7-point Likert-type scale, anchored with ‘1 = strongly disagree’ and ‘7 = strongly agree’ (38). The total scores on the subscales vary from 6 to 42 for WIF, and from 5 to 35 for FIW. The higher the score, the stronger the degree of work–family conflict. The reliability coefficients for the two subscales and the overall scale in our study were 0.968, 0.728, and 0.896, respectively.

#### 3.4.4 Emotional exhaustion

The level of emotional exhaustion among participants was evaluated with the 6-item Emotional Exhaustion Scale (EES) (39), derived from the Maslach Burnout Inventory (40). The scale is well-recognized for its extensive application and validation within the Chinese context (41). A representative item from the scale is, ‘I feel emotionally drained from my work,’ with response options that range on a 5-point Likert scale from ‘1 = strongly disagree’ to ‘5 = strongly agree’ (39). The aggregate score on the EES varies from 6 to 30, with higher scores denoting greater emotional exhaustion. The EES displayed satisfactory reliability in the present study, as indicated by a Cronbach’s alpha coefficient of 0.957.

### 3.5 Data collection

The data collection for this study was conducted from January to April 2023, utilizing a convenience sampling method. Initially, the principal investigator reached out to the heads of nursing departments in targeted hospitals to gain their consent. Subsequently, a QR code, which linked to the survey hosted on www.wjx.cn, was provided to these department heads for distribution to potentially eligible participants in their hospitals. Participation in the study was at the discretion of each individual respondent. To ensure response uniqueness, the questionnaire was configured to allow only one submission per IP address. The questionnaire was designed in a page-flipping format, requiring participants to respond to all questions online and complete the entire survey before submission.

### 3.6 Statistical analysis

We utilized IBM SPSS Statistics 26 along with Hayes’s PROCESS v4.2 Macro (Model 4) within IBM SPSS to perform data analysis. Initially, Harman’s one-factor method was applied to test for common method bias (42). Subsequently, we performed descriptive statistics, *t*-tests, analysis of variance (ANOVA), and Pearson correlation analysis. Continuous variables were described using mean ± standard deviation (SD), while categorical variables were presented in terms of frequency and percentage. The *t*-tests and ANOVA were used to investigate the differences in the level of emotional exhaustion among participants with diverse general characteristics. Bivariate Pearson correlation coefficients were calculated to assess correlations between impostor phenomenon, work–family conflict, and emotional exhaustion. Finally, we proceeded with the mediation effect analysis. The bias-corrected bootstrapping technique with 5,000 bootstrap samples was used to compute the 95% confidence intervals (CI). A statistically significant mediating effect was confirmed if the upper limit and the lower limit of a 95% CI excluded zero (43).

## 4 Results

A total of 4,088 nurses participated in the survey by completing the questionnaires. The final sample for analysis excluded respondents who either provided identical answers to a series of questions or completed the survey in under 180 s (minimum time required for careful completion). The number of valid questionnaires stood at 3977, yielding an effective response rate of 97.3%. The Harman’s one-factor test identified 6 factors with eigenvalues exceeding 1. The first factor accounted for 37.97% of the total variance (< 40%), thus indicating no serious common method bias in this study (42).

### 4.1 Descriptive statistics and group differences

The demographic characteristics of the participants are provided in Table 1. Females represented a significant 95.2% (*n* = 3,786) of the study population, and approximately half of the participants were aged 29–36 years. Among all participants, 71.8% (*n* = 2,856) were married. Only a small percentage (0.4%; *n* = 16) held a graduate degree or higher. Work experience among the participants was fairly evenly distributed, and most possessed junior nursing titles, with 62.9% of them (*n* = 2,503) self-rating their socioeconomic status as being in the middle tier of the overall social hierarchy.

**Table 1:** Demographics of the participating nurses and group differences in emotional exhaustion (*N* = 3,977).

Characteristics	*n* (%)	Mean ± SD	*t/F*	*p*
Gender			−1.532	0.126
Male	191 (4.8%)	16.68 ± 6.67		
Female	3,786 (95.2%)	17.43 ± 6.59		
Age (years)			13.845	< 0.001
≤ 28	1,037 (26.1%)	16.41 ± 6.67		
29–36	1951 (49.1%)	17.79 ± 6.32		
37–44	642 (16.1%)	18.12 ± 6.35		
≥ 45	347 (8.7%)	16.76 ± 6.29		
Marital status^†^			−2.334	0.020
Single	1,121 (28.2%)	17.00 ± 6.58		
Married	2,856 (71.8%)	17.55 ± 6.60		
Educational level			12.953	< 0.001
Junior college and below	1761 (44.3%)	16.81 ± 6.82		
Undergraduate	2,200 (55.3%)	17.87 ± 6.50		
Postgraduate or above	16 (0.4%)	16.13 ± 6.27		
Working experience (years)			14.384	< 0.001
1–5	980 (24.6%)	16.21 ± 6.62		
6–10	1,033 (26.0%)	17.73 ± 6.77		
11–15	1,128 (28.4%)	17.92 ± 6.51		
≥ 16	836 (21.0%)	17.65 ± 6.32		
Professional title			5.066	0.006
Junior	2,865 (72.0%)	17.19 ± 6.73		
Intermediate	908 (22.8%)	17.85 ± 6.13		
Senior	204 (5.1%)	18.20 ± 6.56		
Socioeconomic status^‡^			29.589	< 0.001
Lower	933 (23.5%)	18.47 ± 6.83		
Middle	2,503 (62.9%)	17.35 ± 6.37		
Upper	541 (13.6%)	15.75 ± 6.90		

^† Single indicated separated, divorced, widowed, or never married, and married indicated married or partnered.^

^‡ Socioeconomic status indicated lower tier for choices between 1 ~ 3, middle tier for 4 ~ 7, and upper tier for 8 ~ 10.^

The analysis of group differences in emotional exhaustion of the participants based on their demographic characteristics, as shown in Table 1, revealed significant differences in emotional exhaustion levels across different groups, with the exception of gender. Specifically, higher levels of emotional exhaustion were observed in nurses aged between 29 to 36 and 37 to 44 years, those who were married, possessed an undergraduate degree, with over 6 years of work experience, held an intermediate or senior nursing title, and those from lower or middle socioeconomic tiers.

### 4.2 Bivariate Pearson correlation analyses

Table 2 illustrates means, SD, composite reliability (CR) (44), average variance extracted (AVE), and bivariate Pearson correlations for the examined variables. The CR and AVE values for the variables exceeded their respective thresholds of 0.7 and 0.5, thus confirming the reliability and validity of the measurement scales utilized. Our analysis revealed a positive correlation between impostor phenomenon and WIF (*r* = 0.405, *p* < 0.01), FIW (*r* = 0.372, *p* < 0.01), and emotional exhaustion (*r* = 0.536, *p* < 0.01). A positive correlation was also observed between WIF and both FIW (*r* = 0.392, *p* < 0.01) and emotional exhaustion (*r* = 0.690, *p* < 0.01). Moreover, the relationship between FIW and emotional exhaustion was positively correlated (*r* = 0.409, *p* < 0.01). Given the statistically significant bivariate correlations among the variables specified in the hypothesized pathways, analysis of subsequent mediation effects was pursued.

**Table 2:** Psychometric indicators of the measurement scales and bivariate Pearson correlation analyses.

	CR	AVE	Mean ± SD	IP	WIF	FIW	EE
Impostor phenomenon (IP)	0.948	0.648	28.01 ± 13.85	1			
Work interfering with family (WIF)	0.973	0.858	24.94 ± 10.76	0.405^**^	1		
Family interfering with work (FIW)	0.843	0.561	14.87 ± 6.20	0.372^**^	392^**^	1	
Emotional exhaustion (EE)	0.965	0.822	17.39 ± 6.60	0.536^**^	690^**^	0.409^**^	1

CR, composite reliability; AVE, average variance extracted.

^**^At the 0.01 level (two tails), the correlation is significant.

### 4.3 Parallel mediation analyses

#### 4.3.1 The direct effects of impostor phenomenon on emotional exhaustion

Multiple linear regression analyses controlling for significant demographic variables are detailed in Table 3. The initial univariate analysis showed that impostor phenomenon positively predicted WIF (𝛽 = 0.302, *p* < 0.001), FIW (𝛽 = 0.167, *p* < 0.001), and emotional exhaustion (𝛽 = 0.250, *p* < 0.001). Subsequently, including WIF and FIW as independent variables in the regression model, the multivariate analysis demonstrated a reduction in the predictive power of impostor phenomenon on emotional exhaustion, with the regression coefficient dropping from 0.250 to 0.134, but still maintaining statistical significance (*p* < 0.001). Therefore, Hypothesis 1 was supported by our data.

**Table 3:** Results of multiple linear regression analysis.

Variables	Work interfering with family (WIF)			Family interfering with work (FIW)			Emotional exhaustion (EE)		
	*Coeff.*	*SE*	*p*	*Coeff.*	*SE*	*p*	*Coeff.*	*SE*	*p*
Constant	16.041	0.921	<0.001	12.715	0.538	<0.001	3.820	0.445	<0.001
IP	0.302	0.011	<0.001	0.167	0.007	<0.001	0.134	0.006	<0.001
WIF	—	—	—	—	—	—	0.328	0.007	<0.001
FIW	—	—	—	—	—	—	0.103	0.013	<0.001
Age	−0.781	0.320	0.015	−0.223	0.187	0.235	0.137	0.143	0.337
Marital status	−0.267	0.396	0.501	−0.279	0.231	0.229	−0.015	0.177	0.934
Working experience	0.627	0.252	0.013	0.526	0.148	<0.001	0.166	0.113	0.142
Professional title	0.335	0.355	0.346	−1.341	0.208	<0.001	0.310	0.160	0.052
Educational level	2.024	0.320	<0.001	−0.735	0.187	<0.001	−0.177	0.144	0.219
Socioeconomic status	−1.384	0.261	<0.001	0.029	0.153	0.85	−0.358	0.117	0.002
*R* ^2^	0.181			0.157			0.566		
*F*-value	125.005			105.329			574.365		
*p*	<0.001			<0.001			<0.001		

SE, standard error; IP, impostor phenomenon.

#### 4.3.2 The parallel mediating effects of WIF and FIW

**Figure 1:**
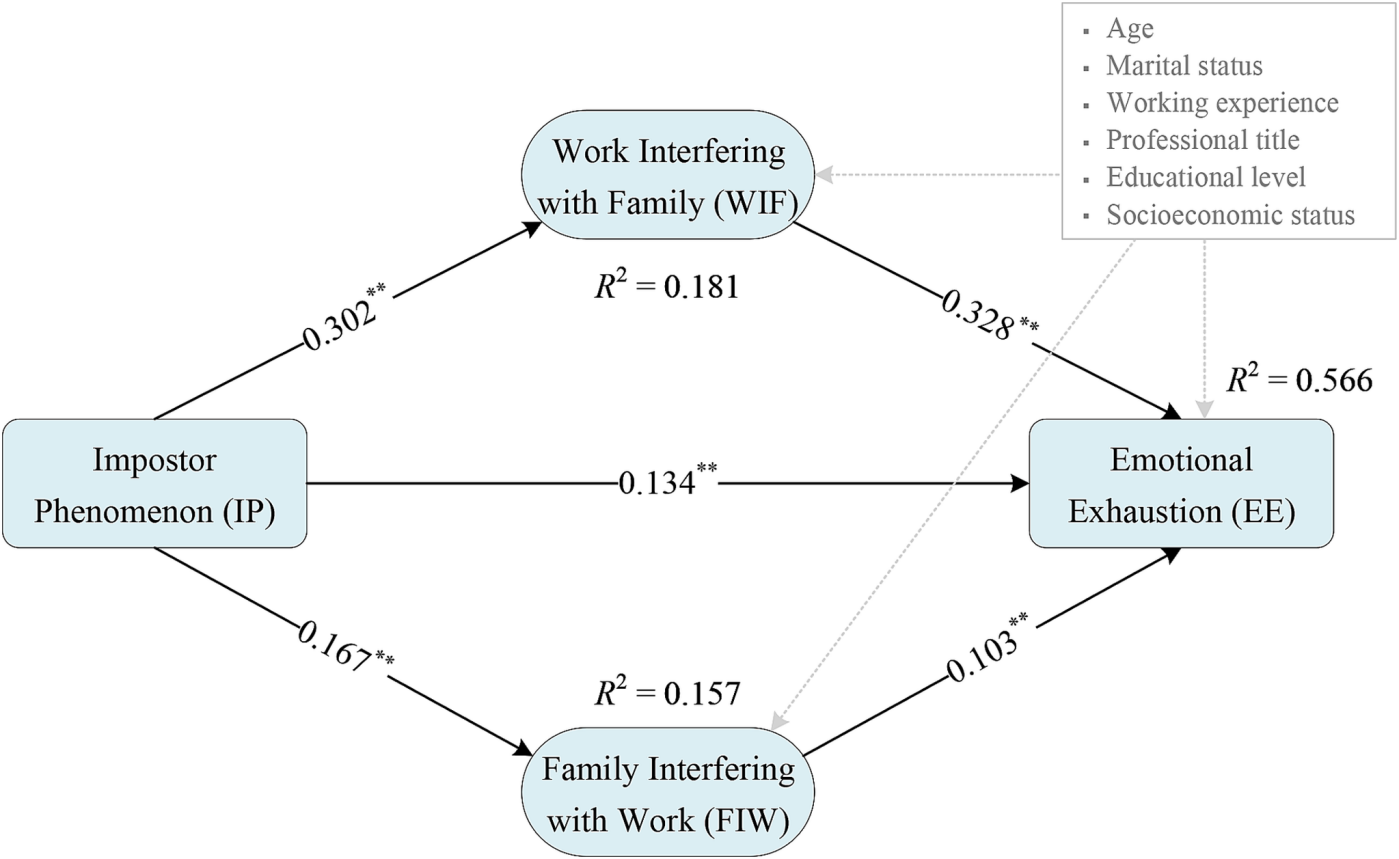
The parallel mediation model with path coefficients for emotional exhaustion. Age, marital status, working experience, professional title, educational level, and socioeconomic status were controlled as confounding factors. *R*^2^ representing the proportion of variation in each dependent variable that can be explained in the model.

The results of the parallel mediation effects analysis using the bias-corrected bootstrapping procedure are illustrated in Figure 1 and Table 4. This analysis revealed that in the model pathway from impostor phenomenon to emotional exhaustion mediated by WIF, the indirect effect was 0.099 (95% CI [0.090 to 0.109]). Similarly, the pathway mediated by FIW showed an indirect effect of 0.017 (95% CI [0.012 to 0.022]). The Bootstrap 95% CIs for both pathways did not cross zero, confirming the significance of these paths. The effect sizes represented 39.6 and 6.8% of the total effect, respectively, for the WIF and FIW mediated pathways. Thus, Hypothesis 2 was verified, affirming that WIF and FIW acted as parallel mediators in the relationship between impostor phenomenon and emotional exhaustion.

**Table 4:** Results of parallel mediation effects analysis with bias-corrected bootstrapping procedure.

Model pathways	Effect	Effect ratio	*SE*	Bootstrap 95% CI	
				Lower	Upper
Total effect	0.250	—	0.006	0.237	0.262
Direct effect: IP → EE	0.134	53.6%	0.006	0.122	0.145
Indirect effect 1: IP → WIF → EE	0.099	39.6%	0.005	0.090	0.109
Indirect effect 2: IP → FIW → EE	0.017	6.8%	0.003	0.012	0.022
Diff (Indirect effect 1–2)	0.082		0.006	0.073	0.096

SE, standard error; CI, confidence interval; IP, impostor phenomenon; EE, emotional exhaustion; WIF, work interfering with family; FIW, family interfering with work. Significant demographic characteristics (age, marital status, working experience, professional title, educational level, and socioeconomic status) were controlled for as confounding factors.

#### 4.3.3 Comparison of the mediating effects of WIF and FIW

The difference in the mediating effects of WIF and FIW was 0.082 (95% CI [0.073 to 0.096]), indicating that the mediating effect of WIF on the relationship between impostor phenomenon and emotional exhaustion was stronger than the mediating effect of FIW. In comparison to FIW, impostor phenomenon had a greater influence on emotional exhaustion through the mediation of WIF. Accordingly, Hypothesis 3 was substantiated by the finding.

## 5 Discussion

The present study explored the relationship between impostor phenomenon and emotional exhaustion among a large multicenter sample of Chinese nurses and examined the parallel mediating role of bi-directional work–family conflict. The findings confirmed our first hypothesis, revealing a direct positive association between impostor phenomenon and emotional exhaustion. Furthermore, the results supported our second and third hypotheses, indicating that WIF and FIW parallelly mediated the pathway linking impostor phenomenon to emotional exhaustion, with WIF exhibiting a more pronounced mediating role compared to FIW. This research represents one of the pioneering efforts to uncover the potential mechanisms underlying the association between impostor phenomenon and emotional exhaustion within the Chinese nursing profession. By elucidating the mediating pathways, our study offers valuable insights for nursing managers to develop targeted interventions aimed at enhancing nurses’ psychological well-being, ultimately improving patient care outcomes and organizational performance.

The first main finding identified that impostor phenomenon had a direct positive effect on emotional exhaustion among nurses, which aligns with previous studies conducted in various populations, including healthcare professionals (9, 12, 13). This direct effect can be attributed to the behavioral characteristics associated with impostorism, such as perfectionism, overworking, and self-criticism (9). Individuals with high levels of impostorism often set excessively high standards for themselves, experience a constant fear of failure or being exposed as frauds, and tend to discount their accomplishments and attribute their success to external factors such as luck or timing (10, 11). Consequently, they may invest a disproportionate amount of time and energy into their work to prove their competence and avoid detection, leading to physical and emotional depletion over time (11). In the context of nursing, the high-stakes nature of the profession, coupled with the intense emotional demands and heavy workload, may amplify the impact of impostor phenomenon on emotional exhaustion (15). The current study’s findings contribute to the growing body of research highlighting the detrimental effects of impostor phenomenon on the mental well-being of nurses, particularly in the Chinese context, underscoring the importance of addressing this psychological vulnerability to promote their emotional well-being and resilience. This finding suggests that healthcare organizations should implement regular psychological screening and support programs specifically targeting impostor phenomenon among nurses, and nurse educators could incorporate impostor phenomenon awareness into professional development initiatives to help nurses better recognize and manage these feelings.

In addition to the direct effect, our study also indicated significant indirect effects of impostor phenomenon on emotional exhaustion, with bi-directional work–family conflict acting as parallel mediators. The indirect effect through WIF can be explained by the behavioral patterns characterizing impostorism, such as overworking and prioritizing occupational demands to validate competence and capability (10). This may lead to a neglect of family responsibilities and heightened WIF, which, in turn, contributes to emotional exhaustion, as the strain of managing competing work and family demands can deplete emotional resources and lead to feelings of overwhelm and burnout. Simultaneously, the indirect effect through FIW can be ascribed to the psychological distress and diminished self-efficacy linked to impostorism (9, 10). The chronic self-doubt, anxiety, and fear of being exposed as incompetent may spill over into the family domain, resulting in increased FIW. Moreover, individuals affected by impostorism may struggle to derive a sense of accomplishment from their personal lives (9), leading to increased FIW and, consequently, exacerbating emotional exhaustion. This is because the burden of attending to family responsibilities while grappling with internal insecurities can drain emotional resources and result in feelings of inadequacy and burnout (14, 29). The findings are consistent with previous research demonstrating the associations between impostor phenomenon, work–family conflict, and emotional exhaustion in other occupational contexts (12, 28, 29). In contrast, the present study extends the existing literature by examining the specific roles of WIF and FIW as parallel mediators and by focusing on the nursing profession in the Chinese context, which has received limited attention in this regard.

Moreover, the stronger mediating effect of WIF compared to FIW in the relationship between impostor phenomenon and emotional exhaustion was observed in our study. This finding can be attributed to the high emotional labor and intense work pressure inherent to the healthcare context in China (45, 46), which may exacerbate the spillover of work-related stress into the family domain. Apart from that, deeply ingrained cultural values of collectivism, respect for authority, and “face-saving” in Chinese society may intensify the pressure on nurses to maintain a flawless image and avoid mistakes in the workplace, thereby increasing their vulnerability to WIF. Such occupational and cultural challenges uniquely predispose Chinese nurses to the detrimental effects of impostorism, further amplifying the impact of WIF on emotional exhaustion. Our study is novel in comparing the mediating effects of WIF and FIW in a large multicenter sample of Chinese nurses, providing insights into the distinct challenges faced by this population and emphasizing the importance of tailoring interventions to address work–family conflict, particularly WIF, in this cultural context. Accordingly, nursing managers should prioritize strategies that help nurses maintain a healthy work-life balance to foster a more resilient and emotionally healthy nursing workforce. Specific interventions could include implementing structured mentorship programs, establishing flexible scheduling options, and developing clear policies limiting overtime work. Organizations should also consider providing practical support services such as childcare assistance and regular professional development workshops focusing on self-efficacy enhancement. These targeted interventions, particularly addressing work interference with family, are crucial given the unique cultural and occupational challenges faced by Chinese nurses.

## 6 Limitations

This study presents several limitations worth noting. First, relying solely on quantitative methods may not fully capture the complexities of nurses’ experiences. Future research could integrate qualitative approaches to provide a more comprehensive understanding of the studied phenomena. Second, the cross-sectional design precludes the establishment of causal relationships among the variables, necessitating longitudinal studies to elucidate the causal associations and temporal dynamics. Third, the sample’s limited representation of male nurses and those with postgraduate education, given their potential impact on the impostor phenomenon, work–family conflict, and emotional responses, calls for further investigation into how these demographic factors influence the studied variables. Fourth, the study’s focus on the relationship between the impostor phenomenon and emotional exhaustion, mediated by work–family conflict, may disregard other relevant mediators or moderators, warranting further exploration of additional pathways and potential factors influencing the associations. Finally, as the multicenter study focused solely on Chinese nurses, caution should be exercised when interpreting and applying the findings to other cultural contexts, underscoring the importance of conducting larger-scale replications in diverse settings to establish generalizability.

## 7 Conclusion

The current study reveals that impostor phenomenon not only directly affects emotional exhaustion but also exerts parallel mediation effects through bi-directional work–family conflict, with WIF exerting a stronger mediating effect than FIW. The findings elucidate the complex interplay between impostor phenomenon, an intrapersonal psychological factor, and work–family conflict, an interpersonal stressor, in contributing to emotional exhaustion among Chinese nurses. T These findings underscore the imperative for healthcare organizations to implement dual-focused interventions that address both impostor phenomenon and work-family dynamics, with particular emphasis on mitigating work-to-family interference. Such evidence-based approaches could enhance nurses’ psychological well-being and workforce sustainability in healthcare settings.

## Data Availability

The original contributions presented in the study are included in the article/supplementary material, further inquiries can be directed to the corresponding authors.

## Footnotes

^1^ https://www.gov.cn/zhengce/2016-10/25/content_5124174.htm

^2^ https://www.gov.cn/zhengce/zhengceku/2022-05/09/content_5689354.htm

^3^ https://www.who.int/publications-detail-redirect/9789240003279

^4^ https://www.aacn.org/blog/imposter-syndrome-in-nursing-a-barrier-to-personal-growth
